# Supplementary material for: Spatial and temporal control of lysis by the lambda holin
Source: mBio. 2023 Dec 21;15(2):e01290-23. doi: 10.1128/mbio.01290-23 (PMC10865782; doi:10.1128/mbio.01290-23)
Supplement: Supplemental file — Fig. S1 and S2, Tables S1 and S2, and movie legends. [file mbio.01290-23-s0001.docx]

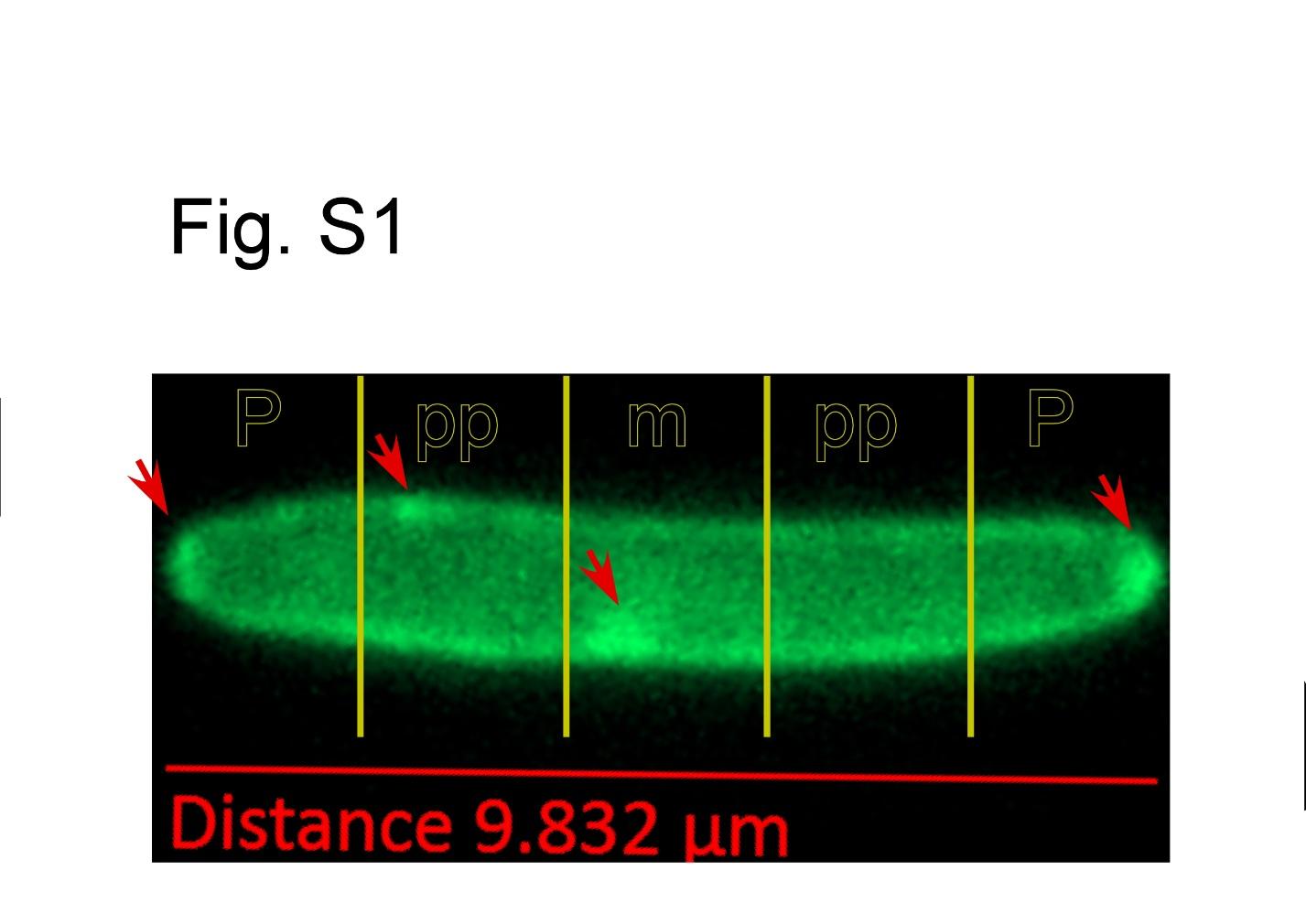


## Figure S1. Assigning rafts to subcellular compartments. P=polar, pp=peripolar, m=midcell.


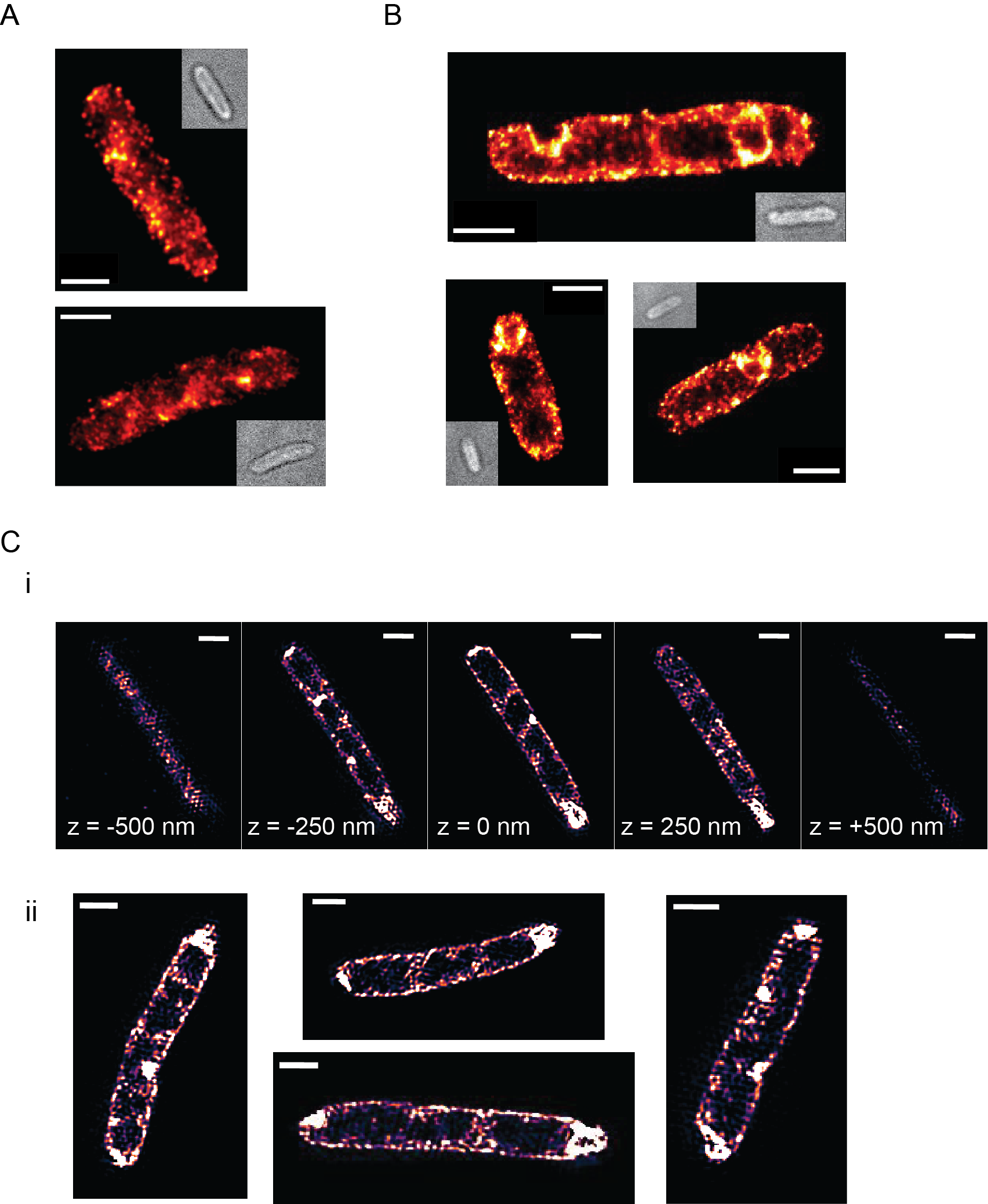


## Figure S2. Immuno-STORM imaging of S105 and S105-A52I

Superresolution imaging of S105 and the A52I non-lytic holin mutant. Representative STORM images of (A) WT holin at 50 min post-induction of MG1655(λ*R_am_*), and (B) λ900 ΔSR lysogens induced for *S105_A52I_* imaged at 50 min post-induction, inset images are brightfield images of the cells; each superresolution image contains fitted spots represented as two-dimensional Gaussians, normalized against the total intensity, scale bars represent one micron. (C) Reconstructed SIM images of the A52I non-lytic holin mutant at 50 min post-induction, with (i) showing a montage of a single cell in the z-dimension demonstrating an invaginated holin raft with (ii) showing representative images of rafts at the mid-cell plane; scale bars represent one micron.

**Table S1. Lysis of spanin mutant cells treated with EDTA prior to lysis. The number of blebs detected per cell, subcellular localization of blebs and lysis morphology are described per cell. P = polar; pp = parapolar; m = midcell. “-” indicates blebs were not detected.**

| Cell | Number of blebs | Subcellular location | Lysis morphology |
| --- | --- | --- | --- |
| 1 | 1 | P | Rod |
| 2 | 5 | P, P, P, pp, m | Sphere |
| 3 | 1 | P | Rod |
| 4 | 2 | P, P, m | Rod |
| 5 | 1 | P | Rod |
| 6 | 1 | P | Rod |
| 7 | 3 | P, P, m | Rod |
| 8 | 3 | P, P, P | Rod |
| 9 | 2 | P, P | Rod |
| 10 | 3 | P, P, P | Rod |
| 11 | 2 | P, P | Rod |
| 12 | 3 | P,P, pp | Rod |
| 13 | 4 | P, P, pp, pp, | Rod |
| 14 | 3 | P, P, pp | Rod |
| 15 | 2 | P, P, | Rod |
| 16 | - | - | Sphere |
| 17 | 3 | P, P, m | Rod |
| 18 | 2 | P, P, | Rod |
| 19 | 3 | P, P, m | Rod |
| 20 | - | - | Sphere |
| 21 | 2 | P, P, | Rod |
| 22 | 2 | P, P, | Rod |
| 23 | 1 | P | Rod |
| 24 | - | - | Sphere |
| 25 | - | - | Sphere |
| 26 | 3 | P, pp, m | Rod |
| 27 | 3 | P, P, P | Rod |
| 28 | 2 | P, P, | Rod |
| 29 | - | - | Sphere |
| 30 | - | - | Sphere |
| 31 | 3 | P, P, pp | Rod |
| 32 | 2 | P, pp | Rod |
| 33 | - | - | Sphere |
| 34 | 4 | P, P, pp, m | Rod |
| 35 | - | - | Sphere |
| 36 | 1 | P | Rod |
| 37 | - | - | Sphere |
| 38 | 3 | P, P, m | Rod |
| 39 | - | - | Sphere |
| 40 | 3 | P, P, pp | Rod |
| 41 | 3 | P, P, pp | Rod |
| 42 | 2 | P, P | Rod |
| 43 | - | - | Sphere |
| 44 | - | - | Sphere |

**Table S2. Lysis of cells expressing *S105-GFP.* The table is ordered by the monitoring time before lysis. Time is reported in seconds. Breach site and raft locations are indicated by P=polar, m= midcell, pp =parapolar. NA. = uncertain because the parameter could not be detected. The “time raft to lysis” measures the seconds between raft formation and lysis. “time raft lost” is the interval of time from raft disappearance to lysis.**

| cell | time to lyse | lysis site | # of rafts | raft location | time raft appears | time raft gone | time raft to lysis | | Time raft lost | | phase light raft | | raft flicker | | rafts predict  lysis site | |
| --- | --- | --- | --- | --- | --- | --- | --- | --- | --- | --- | --- | --- | --- | --- | --- | --- |
| 1 | 1 | P | NA | NA | NA | NA | NA | | NA | | NA | | NA | | NA | |
| 2 | 1 | m | NA | NA | NA | NA | NA | | NA | | NA | | NA | | NA | |
| 3 | 1 | m | NA | NA | NA | NA | NA | | NA | | NA | | NA | | NA | |
| 4 | 41 | m | NA | NA | NA | NA | NA | | NA | | NA | | NA | | NA | |
| 5 | 57 | P | 1 | P | 0 | 19 | NA | | 38 | | - | | - | | + | |
| 6 | 80 | P | 2 | P, P | 0 | 21 | NA | | 59 | | - | | - | | + | |
| 7 | 85 | pp | 4 | P, P, pp, m | 0 | 71 | NA | | 14 | | - | | - | | + | |
| 8 | 86 | m | 1 | P | 0 | 43 | NA | | 43 | | - | | - | | - | |
| 9 | 121 | m | 2 | P, P | 57 | 100 | 64 | | 21 | | + | | - | | - | |
| 10 | 126 | P | 2 | P, P | 17 | 59 | 109 | | 67 | | + | | + | | + | |
| 11 | 137 | P | 2 | P, pp | 0 | NA | NA | | NA | | + | | - | | + | |
| 12 | 141 | P | 3 | P, P, m | 64 | 122 | 77 | | 19 | | + | | - | | + | |
| 13 | 150 | m | 2 | P, P | 20.5 | 139 | 129.5 | | 11 | | + | | - | | - | |
| 14 | 156 | P | 2 | P, P | 64 | 145 | 92 | | 11 | | + | | - | | + | |
| 15 | 161 | m | 2 | P, m | 52 | 145 | 109 | | 16 | | + | | - | | + | |
| 16 | 162 | P | 3 | P, pp, m | 0 | NA | NA | | NA | | + | | - | | + | |
| 17 | 166 | P | 2 | P, pp | 69 | 94 | 97 | | 72 | | + | | - | | + | |
| 18 | 170 | m | 2 | P, P | 72 | 132 | 98 | | 38 | | - | | - | | - | |
| 19 | 170 | P | 1 | P | 0 | 157 | NA | | 13 | | + | | - | | + | |
| 20 | 179 | pp | 3 | P, P, pp | 77 | 137 | 102 | | 42 | | - | | + | | - | |
| 21 | 179 | P | 5 | P, P, P, pp, pp | 104 | 165 | 75 | | 14 | | + | | - | | + | |
| 22 | 185 | P | 3 | P, P, pp | 93 | 167 | 92 | | 18 | | - | | - | | - | |
| 23 | 186 | P | 2 | P, P | 68 | 118 | 118 | | 68 | | + | | - | | + | |
| 24 | 191 | P | 2 | P, P | 95 | 158 | 96 | | 33 | | + | | + | | + | |
| 25 | 195 | pp | 2 | P, P | 130 | 165 | 65 | | 30 | | + | | - | | - | |
| 26 | 196 | P | 2 | P, P | 116 | 166 | 80 | | 30 | | + | | - | | + | |
| 27 | 203 | P | 2 | P, pp | 82 | 150 | 121 | | 53 | | - | | - | | + | |
| 28 | 205 | m | 2 | P, P | 96 | 136 | 109 | | 69 | | + | | - | | - | |
| 29 | 211 | m | 1 | P | 133 | 190 | 78 | | 21 | | + | | - | | - | |
| 30 | 214 | P | 2 | P, P | 66 | 177 | 148 | | 37 | | + | | + | | + | |
| 31 | 215 | m | 5 | P, P, P, pp, m | 119 | NA | 96 | | NA | | - | | - | | + | |
| 32 | 219 | pp | 2 | pp, pp | 140 | 208 | 79 | | 11 | | + | | - | | - | |
| 33 | 221 | pp | 4 | P, P, pp, pp, | 134 | 175 | 87 | | 46 | | + | | - | | + | |
| 34 | 222 | pp | 4 | P, pp, pp, m | 152 | 208 | 70 | | 14 | | - | | - | | + | |
| 35 | 223 | P | 1 | P | 124 | 189 | 99 | | 34 | | + | | - | | + | |
| 36 | 224 | P | 2 | pp, m | 153 | 206 | 71 | | 18 | | + | | - | | - | |
| 37 | 225 | pp | 4 | P, P, pp, pp | 135 | 175 | 90 | | 50 | | + | | - | | + | |
| 38 | 227 | m | 4 | P, P, m, m | 143 | NA | 84 | | NA | | + | | - | | + | |
| 39 | 252 | P | 2 | P, P | 108 | 164 | 144 | | 88 | | + | | - | | + | |
| 40 | 252 | pp | 2 | P, P | 166 | 225 | 86 | | 27 | | + | | + | | - | |
| 41 | 259 | P | 2 | P, P | 167 | NA | 92 | | NA | | + | | - | | + | |
| 42 | 265 | pp | 1 | P | 186 | 229 | 79 | | 36 | | + | | - | | - | |
| 43 | 280 | P | 2 | P, P | 178 | 263 | 102 | | 17 | | + | | - | | + | |
| 44 | 282 | P | 1 | P | 193 | 230 | 89 | | 52 | | - | | - | | + | |
| 45 | 292 | PP | 4 | P, P, pp, m | 215 | 252 | 77 | | 40 | | + | | - | | - | |
| 46 | 303 | P | 3 | P, P, m | 222 | 271 | 81 | | 32 | | - | | - | | + | |
| 47 | 374 | P | 2 | P, P | 289 | 329 | 85 | | 45 | | - | | - | | + | |
| 48 | 392 | P | 1 | P | 246 | 325 | 146 | | 67 | | + | | - | | - | |
| 49 | 472 | P | 1 | P | 401 | 444 | 71 | | 28 | | + | | + | | + | |
| 50 | 478 | PP | 3 | P, P, m | 422 | 455 | 56 | | 23 | | + | | - | | - | |
|  | | | | | | | | | | | | | | | | |
| Average | | | 2.3 | Average | | | | 93.4 | | 35.7 | | 33/46 | | 6/46 | | 30/46 |
| Standard Deviation | | | 1.1 | Standard Deviation | | | | 22.2 | | 20.0 | |  | |  | | 65% |
|  | | | n=46 |  | | | | n=39 | | n=41 | |  | |  | |  |

1. Supplemental Movie - Movie 1
   two clips: 1st= polar lysis morphology of cell infected by wt lambda, 2nd= lysis is blocked when for spanin amber lambda mutants; spherical conversion observed
2. Supplemental Movie - Movie 2 
   Expression of a modified lambda endolysin targeted for secretion into the periplasm. Cells gradually convert to spherical morphology as the peptidoglycan is degraded.
3. Supplemental Movie - Movie 3 two clips. Lysis morphology examples when cells infected with spanin-amber mutant phage are treated with EDTA just prior to lysis
4. Supplemental Movie - Movie 4 
   Less frequently, lambda-infected cells that are exposed to EDTA prior to lysis exhibit a rounding (not polar) lysis morphology.
5. Supplemental Movie - Movie 5 
   Time-lapse fluorescence microscopy of lambda-infected cells carrying a GFP-tagged holin.
6. Supplemental Movie - Movie 6 
   Time-lapse fluorescence microscopy of lambda-infected cells carrying a GFP-tagged holin. Rafts were sometimes unstable, forming foci, then delocalizing before forming foci again.
7. Supplemental Movie - Movie 7 
   Time-lapse fluorescence microscopy of lambda-infected cells exposed to thioflavin T.
8. Supplemental Movie - Movie 8 
   Time-lapse fluorescence microscopy of lambda-infected cells (carrying spanin amber mutants) exposed to thioflavin T.
9. Supplemental Movie - Movie 9 
   Time-lapse fluorescence microscopy of lambda-infected cells (expressing spanins and secreted endolysin in place of the normal lysis cassette) exposed to thioflavin T.
